# The Association between exposure to COVID-19, internalizing symptoms, and Dispositional Mindfulness in Adolescents: a longitudinal pre- and during-pandemic study

**DOI:** 10.1007/s10578-022-01349-0

**Published:** 2022-04-15

**Authors:** Estíbaliz Royuela-Colomer, Liria Fernández-González, Izaskun Orue, Esther Calvete

**Affiliations:** Department of Personality, Psychological Assessment and Treatment, University of Deusto, Bilbao, Spain

**Keywords:** COVID-19, Dispositional mindfulness, Internalizing symptoms, Adolescents

## Abstract

This study examined the association between contact with COVID-19 and internalizing symptoms in Spanish adolescents, and the moderation and mediation roles of dispositional mindfulness. Adolescents (*N* = 383; 58% female; *M*age = 15.62, *SD* = 1.32) completed measures of dispositional mindfulness (MAAS-A) and internalizing symptoms (DASS-21), other stressors different from COVID-19, and contact with COVID-19 twice, in October 2019 and 2020. Three profiles emerged according to their contact with COVID-19: (1) little/no contact, (2) knowing someone close (outside home) who was infected, hospitalized, or died, and (3) being or someone at home being infected and/or hospitalized. Compared to little/no contact, both contact profiles predicted dispositional mindfulness and anxiety; and profile 2 predicted stress. Dispositional mindfulness mediated the association between both contact profiles and depression and stress. This study suggests that contact with COVID-19 predicts increased internalizing symptoms in adolescents, which could be partially explained by the decrease in mindfulness levels.

Adolescence is a period of developmental changes often associated with increased internalizing symptoms, including depression, anxiety, and stress [1–3]. The COVID-19 pandemic has brought new challenges for adolescents, specifically social isolation, homeschooling, family members being infected with COVID-19, and dealing with difficult situations, such as losing a beloved one [4, 5]. Adolescents possess limited resources to cope with these stressful situations since many coping strategies are still developing through adolescence [6, 7]. In addition, social isolation can have a profound impact since adolescence is an evolutionary period during which the relationship with peers and development beyond the family is particularly relevant [3].

Although it is still early to know with certainty, emerging evidence suggests that the long-term consequences of the COVID-19 pandemic will be especially detrimental for adolescents. For example, several reviews reported an increase in anxiety, depression, post-traumatic stress symptoms, and sleep disturbances among adolescents, particularly in girls [8–11]. However, most of these studies are cross-sectional and often do not include pre-pandemic data for comparison. Thus, more longitudinal research is needed to better understand the long-term effects of COVID-19 on adolescents’ mental health. The main goal of this study was to examine the association between contact with COVID-19 (i.e., having been infected or knowing someone who has been infected, hospitalized, or died) and an increase in internalizing symptoms in Spanish adolescents, controlling for pre-pandemic symptoms. Additionally, this study examines whether dispositional mindfulness (DM)—which has been associated with positive mental health through adolescence [12, 13]—moderates and/or mediates the association between contact with COVID-19 and internalizing symptoms.

## The Impact of COVID-19 on Adolescent Mental Health

Although adolescents are less susceptible than adults to developing severe cases of COVID-19 [14], many experts claim that the impact of the pandemic on adolescents’ mental health will be profound and emphasize the need to conduct more research [4, 15]. Several factors have been recognized to influence adolescents’ mental health during COVID-19. Research has shown that the disruption of social life caused by confinement, quarantine, and social restrictions is associated with loneliness, post-traumatic stress symptoms, confusion, depression, anxiety, irritability, and anger [16–18]. In addition, school closure has been associated with increased anxiety and loneliness [19]. Indeed, adolescents reported that one of the most distressing things about the pandemic was missing contact with their friends [20, 21]. Moreover, lifestyle changes that emerged due to COVID-19 have been associated with an increase in social medial and Internet use, which in turn has been suggested as a risk factor for distress among adolescents during the pandemic [8].

Adolescents may also experience distress due to the COVID-19 pandemic itself. For example, one study found that fear of a family member being infected with COVID-19 was a risk factor for negative mental symptoms among adolescents [22]. Similarly, a review found that worry, fear about contracting the virus, and stress predicted distress in adolescents [11]. In fact, one study found that knowing a family member or friend who was infected and got very sick or died from COVID-19 was among the most stressful aspects of the pandemic for adolescents [20]. Therefore, it could be that having closer contact with COVID-19 might have caused stress, fear, worry and placed adolescents in difficult situations that they might not have been able to cope with adequately. Furthermore, many of their coping resources for managing stressful situations have been limited (e.g., meeting friends), and greater contact with the coronavirus may have even diminished their protective traits, such as mindfulness.

## Mindfulness and COVID-19

Mindfulness has been defined as “the awareness that emerges through paying attention on purpose, in the present moment, and nonjudgmentally to the experience moment by moment” (p.145) [23]. Mindfulness can be a state, a training, or a disposition that can be promoted through training. DM has been associated with positive mental health through adolescence [24], and it is the focus of the present study. Specifically, a study suggested that DM could be beneficial for adolescents experiencing high perceived life stress, as it is related to reduced emotional and cardiovascular reactivity to stress [25]. Additionally, a considerable amount of evidence suggests that DM moderates the impact of stressors on mental health in adolescents [13, 26]. Related to COVID-19, although there are no previous studies using adolescent samples, a study on adults found that higher DM was associated with less stress, anxiety, depression, worry about the virus, a less negative affect if one gets the virus, and healthier strategies for coping with COVID-19 [27]. Another study of adults found that DM moderated the association between fear of COVID-19 and internalizing symptoms [28].

Furthermore, stressful situations, such as those caused by the current pandemic, could also affect DM levels. In this vein, one study found that post-traumatic stress symptoms from experiencing a tornado longitudinally predicted less DM in adolescents and suggested that traumatic events might influence the capacity to be mindful [29]. Regarding COVID-19, a study including college students reported that DM mediated the association between fear of COVID-19 and anxiety and depressive symptoms [30]. Specifically, the study reported that lower levels of fear of COVID-19 were associated with more DM levels, and DM, in turn, was related to less anxiety and depression symptoms. However, this was a cross-sectional study, and therefore it could not examine the longitudinal effects of COVID-19 on DM levels. To the best of our knowledge, no previous study has examined the relationship between contact with COVID-19, DM, and internalizing symptoms longitudinally using pre-pandemic data in adolescents.

## Current Study

The main goal of this study was to examine the association between COVID-19 contact, DM, and internalizing symptoms (depression, anxiety, and stress) in Spanish adolescents. The main hypotheses were that contact with COVID-19 would predict more internalizing symptoms and that DM would attenuate the association between contact with COVID-19 and the change in internalizing symptoms over time. In addition, it was hypothesized that contact with COVID-19 would predict a decrease in DM, which would explain the increase in internalizing symptoms. Other stressors different from COVID-19, age, and SES were controlled. Furthermore, the model’s sex invariance was tested, as internalizing symptoms are more common among adolescent girls than among boys [1], and girls have a higher possibility of developing emotional problems when exposed to stress during adolescence [31]. In fact, a recent study during COVID-19 showed that adolescent girls experienced a greater decline in mental health compared to boys [20]. Further, recent evidence suggests that DM could be more beneficial for boys than for girls [32]. Finally, a secondary goal was to identify classes of adolescents according to their level of contact with COVID-19.

## Method

### Study Design

This study utilized a two-wave longitudinal design with a one-year time interval between wave one (W1) in October 2019—before the onset of COVID-19—and wave two (W2) in October 2020—after the onset of COVID-19. The ethical committee of the University of Deusto approved the study.

## Procedure

Two schools from Vitoria-Gasteiz (Spain), which participated in a study in 2019 and were selected by convenience sampling, were informed and invited to participate in a follow-up study that involved collecting data on COVID-19. Both schools were private schools that receive public funds (i.e., *colegios concertados*). Only one of the two schools agreed to participate. Once the school principal and teachers agreed to participate, information about the study was provided to families, including informed consent. The students who agreed to participate completed the questionnaires in class at both waves, including demographic data, on their computers using the Qualtrics® online platform. All data were anonymous; each student had a unique code (i.e., participants’ date of birth and parents’ first initial) to match responses between waves. The participants completed a measure of internalizing symptoms and DM at both waves. Additionally, at W2, participants reported their contact with COVID-19 and the number of stressful events (different from COVID-19) that occurred since they completed W1. As compensation for their participation, students received a raffle ticket for a 20€ coupon.

## Participants

The participants were 513 high school students from first year of Compulsory Secondary Education to first baccalaureate (grades 7 to 11) at W1. Of those participants, 455 completed the questionnaires in W1, while 58 were new in W2 (i.e., did not complete the questionnaires in W1, but they did in W2). From the initial sample, 438 agreed to participate at W2, but 55 were excluded because they did not provide data about their contact with COVID-19. All the participants who completed the questionnaires at least at W2 were included in the analyses. Thus, the final sample was composed of 383 students (58% female; *M*_*age*_ = 15.62, *SD*_*age*_ = 1.32 at W2), of which 86.2% (*n* = 330) provided data at both W1 and W2. There were no significant differences in sex between excluded (*n* = 130) and included participants (*n* = 383, *X* ^2^(1, *N* = 513) = 0.54, *p* = .46). There was a significant age difference: excluded participants (*M* = 14.12, *SD* = 1.56) were younger at W1 compared to included participants (*M* = 14.57, *SD* = 1.33, *t*(509) = 3.18, *p* = .002, *d* = 0.31). The reason for the age difference is that a complete class that participated at W1 was not able to participate at W2. Following the guidelines of The Spanish Society of Epidemiology and the Spanish Society of Family and Community Medicine [33], we used parental occupation and education to determine participants’ socioeconomic status (SES). The SES distribution was as follows: 6% low status, 11.5% low-medium status, 33.8% medium status, 41.4% medium-high status, and 7.3% high status.

## Measures

### Internalizing Symptoms

The Depression, Anxiety and Stress Scale-21 Items (DASS-21) [34] is composed of 21 items divided into three subscales, with seven items each, that measure low positive affect (depression), physiological hyperarousal (anxiety), and negative affect (stress). Sample items include “I couldn’t seem to experience any positive feeling at all,” “I felt I was close to panic,” “I tended to over-react to situations.” Participants had to rate each item, selecting a number that indicates to what degree this statement or experience has occurred to them during the past week using a four-point scale, ranging from 0 (*did not apply to me at all*) to 3 (*applied to me very much or most of the time*). Previous studies reported good psychometric properties in young Hispanic samples [35, 36]. In this study, Cronbach’s α of the total DASS score was.93 both at W1 and W2, and for the subscales, α was 0.87 and 0.88 (depression), 0.82 and 0.84 (anxiety), 0.81 and 0.81 (stress) at W1 and W2, respectively.

### Stressors

The participants completed the Life Stress Questionnaire (LSQ) [37]. The version used in this study assesses the occurrence of 12 common stressors since the beginning of the pandemic, including financial problems at home, academic problems, health problems (different from COVID-19), changes at home responsibilities, death of a close relative (not from COVID-19), parents’ divorce/separation, work problems at home, change of house/neighborhood, significant health problems in relatives/friends (different from COVID-19), arguments with friends/partner break-up, and problems/arguments with family members. A composite score was generated by summing all the items; the variable ranged from 0 to 12. The equivalence between the Spanish and the English versions of this measure has been demonstrated [38].

### COVID-19 Contact

The participants indicated whether, since the beginning of the pandemic, the following six questions—indicating their contact with COVID-19—were true for them: (1) Have you been infected with COVID-19?; (2) Have any of the people you live with been infected with COVID-19?; (3) Have any of the people you live with been hospitalized due to COVID-19?; (4) Has someone close to you (i.e., family, close friends, acquaintances) been infected with COVID-19?; (5) Has someone close to you (i.e., family, close friends, acquaintances) been hospitalized due to COVID-19?; and (6) Has anyone close to you (i.e., family, close friends, acquaintances) died from COVID-19?

### Dispositional Mindfulness

The participants completed the Spanish version of the Mindful Attention Awareness Scale-Adolescents (MAAS-A) [24, 39]. The MAAS-A is a 14-item self-report questionnaire that measures DM as the presence of attention to and awareness of what is occurring in the present moment (e.g., “I find myself preoccupied doing things without paying attention” and “I forget a person’s name almost as soon as I’ve been told it for the first time”). The participants rated each statement using a six-point scale, ranging from 1 (*almost never*) to 6 (*almost always*). Previous studies on Spanish adolescents reported adequate psychometric properties [39]. The present study’s internal consistency was α = 0.81 at W1 and W2.

### Data Analysis

Data were analyzed using IBM SPSS Statistics (Version 26) [40], R (Version 1.3.1056) [41], and MPLUS (Version 8.6) [42]. Little’s MCAR (Missing Completely At Random) test was statistically significant (χ^2^(27) = 51.23, *p* < .001). Therefore, the full information maximum likelihood (FIML) method was employed to manage missingness with R. For the DM variable, 14.6% and 1% of the participants had missing values at W1 and W2, respectively; for the internalizing symptoms variable, 15.1% and 1% had missing values at W1 and W2 respectively; and for the stress variable, 1.6% of the participants had missing values.

Adolescents’ classes based on their contact with COVID-19 were identified using latent class analyses with the maximum likelihood (ML) estimator in MPLUS 8.6 [43]. A one-class model was estimated as a comparative baseline for the following models, including a progressively increasing number of classes. These solutions were evaluated through several indicators: Akaike Information Criterion (AIC), Bayesian Information Criterion (BIC), Sample-Size Adjusted BIC (SSABIC), entropy index, and the parametric bootstrapped likelihood ratio test (BLRT). For AIC, BIC, and SSABIC, lower values indicate a superior fit [44]. Entropy values ≥ 0.80 indicate a good classification of individuals into classes. The BLRT compares the fit between two neighboring class models (i.e., a *k*-class versus a *k*-1-class model). A non-significant *p*-value for a *k-*class solution is considered as support for the *k*-1 class solution [44].Finally, the characteristics of the profiles and the number of participants within each class were also considered.

Path analysis was computed using the R package *Lavaan* [45] and ML. The hypothesized model included cross-sectional associations between all the study variables at W1 (internalizing symptoms, DM, stressors, contact with COVID-19) and W2 (internalizing symptoms, DM), autoregressive paths from internalizing symptoms and DM at W1 to the same variables at W2, and cross-lagged predictive paths from W1 to W2 variables. Additionally, age and SES were included as covariates at W1. Finally, the model included paths from the interaction terms between DM and contact with COVID-19 profiles.

We evaluated the model’s goodness of fit with the comparative fit index (CFI), the Tucker Lewis index (TLI), the root mean square error of approximation (RMSEA), and the standardized root mean square residual (SRMS). Generally, an acceptable fit is indicated by CFI and TLI values greater than 0.95, RMSEA values below 0.06, and SRMS values below 0.08 [46]. Additionally, we conducted a multiple-group analysis to explore the model’s sex invariance. We first tested the full model separately for boys and girls. A more parsimonious model was estimated, including the significant paths in the full model and in boys and girls separately. Then, we tested this model’s configural invariance to demonstrate that the pattern of fixed and free parameters was equivalent across subsamples. Third, the invariance of the longitudinal paths of the model was tested. Finally, bootstrapping (*n* = 5000 samples) was used to test the significance of the indirect effects (i.e., a × b, where a is the path between the predictor and the mediator, and b is the path between the mediator and the outcome). The indirect effect was considered significant at the 0.05 level if the 95% confidence level did not include zero [47].

## Results

### Descriptive Statistics

Of the total sample, 65.3% indicated knowing someone (close friend, acquaintance, or relative) who was infected with coronavirus; 21.1% knew someone hospitalized, and 13.8% reported that someone close to them died due to COVID-19; 14.9% indicated being infected themselves with coronavirus; 19.6% had someone they lived with who was infected, and 2.1% indicated that someone they lived with had been hospitalized. Table 1 shows the descriptive statistics for all the study variables at W1 and W2. There was a significant difference in internalizing symptoms and DM levels from W1 to W2 (*t*(320) = -2.49, *p* = .013, *d* = 0.14 and *t*(322) = 2.69, *p* < .007, *d* = − 0.14, respectively). Specifically, there was an increase in depressive symptoms from W1 to W2, *t*(320) = -3.04, *p* = .003, *d* = 0.18. Table 2 shows the correlation coefficients among the variables. There was a negative correlation between all internalizing symptoms and DM at both waves; stressors were negatively correlated with DM and positively correlated with all internalizing symptoms at both waves. Age was only negatively correlated with DM at W1, and SES was only negatively correlated with stressors and depressive symptoms at W2.

**Table 1:** Descriptive Statistics for all the Study Variables

Variable		Total Sample (*N* = 383)		Class 1 (*n =* 244)		Class 2 (*n =* 72)		Class 3 (*n =* 67)
		*M* (*SD*)		*M* (*SD*)		*M* (*SD*)		*M* (*SD*)
Internalizing Symptoms	W1	0.96 (0.62)*		0.97 (0.64)		0.95 (0.65)*		0.91 (0.49)*
	W2	1.06 (0.65)*		0.98 (0.63)		1.24 (0.66)*		1.17 (0.65)*
Depression	W1	0.83 (0.73)*		0.86 (0.75)		0.81 (0.73)*		0.75 (0.65)*
	W2	0.99 (0.78)*		0.92 (0.77)		1.13 (0.79)*		1.07 (0.78)*
Anxiety	W1	0.84 (0.68)		0.85 (0.71)		0.82 (0.67)*		0.83 (0.53)*
	W2	0.93 (0.72)		0.83 (0.68)		1.11 (0.77)*		1.09 (0.76)*
Stress	W1	1.20 (0.68)		1.21 (0.68)		1.22 (0.72)*		1.15 (0.60)
	W2	1.27 (0.68)		1.19 (0.68)		1.47 (0.68)*		1.35 (0.64)
Dispositional Mindfulness	W1	4.38 (0.78)*		4.41 (0.78)		4.30 (0.82)		4.38 (0.70)*
	W2	4.25 (0.78)*		4.36 (0.78)		4.07 (0.75)		4.07 (0.72)*
Stressors	W1-W2	3.01 (2.24)		2.78 (2.17)		3.51 (2.23)		3.31 (2.43)
*Note.* Class 1: no contact at all or just knowing someone (friend, family, or acquaintance outside home) infected with COVID-19; Class 2: knowing someone (friend, family, or acquaintance outside home) who has been infected, hospitalized, or die with COVID-19; Class 3: having themselves or living with someone who had COVID-19 or has been hospitalized; Wave 1: Data was collected in October 2019; Wave 2: Data was collected in October 2020 *Significant differences between W1 and W2: *p* < .05								

**Table 2:** Correlations Between all the Study Variables

Variables	1	2	3	4	5	6	7	8	9
1. Depression (W1)	-								
2. Anxiety (W1)	0.69**	-							
3. Stress (W1)	0.65**	0.71**	-						
4. Dispositional Mindfulness (W1)	− 0.46**	− 0.48**	− 0.55**	-					
5. Stressors W1-W2	0.24**	0.27**	0.23**	− 0.23**	-				
6. Depression (W2)	0.47**	0.44**	0.35**	− 0.36**	0.37**	-			
7. Anxiety (W2)	0.38**	0.49**	0.37**	− 0.30**	0.39**	0.72**	-		
8. Stress (W2)	0.34**	0.41**	0.46**	− 0.44**	0.41**	0.62**	0.73**	-	
9. Dispositional Mindfulness (W2)	− 0.34**	− 0.36**	− 0.38**	0.60**	− 0.35**	− 0.48**	− 0.49**	− 0.59**	-
10. Age	0.08	− 0.04	0.07	− 0.15**	0.08	− 0.03	− 0.03	0.00	− 0.06
11. Socioeconomic Status	− 0.06	− 0.10	0.02	− 0.09	− 0.20**	− 0.13*	− 0.13*	− 0.06	0.01
*Note*: ^*^ *p* < .05; ^**^ *p* < .01									

## Classes of Adolescents According to Their Contact with COVID-19

**Fig. 1:**
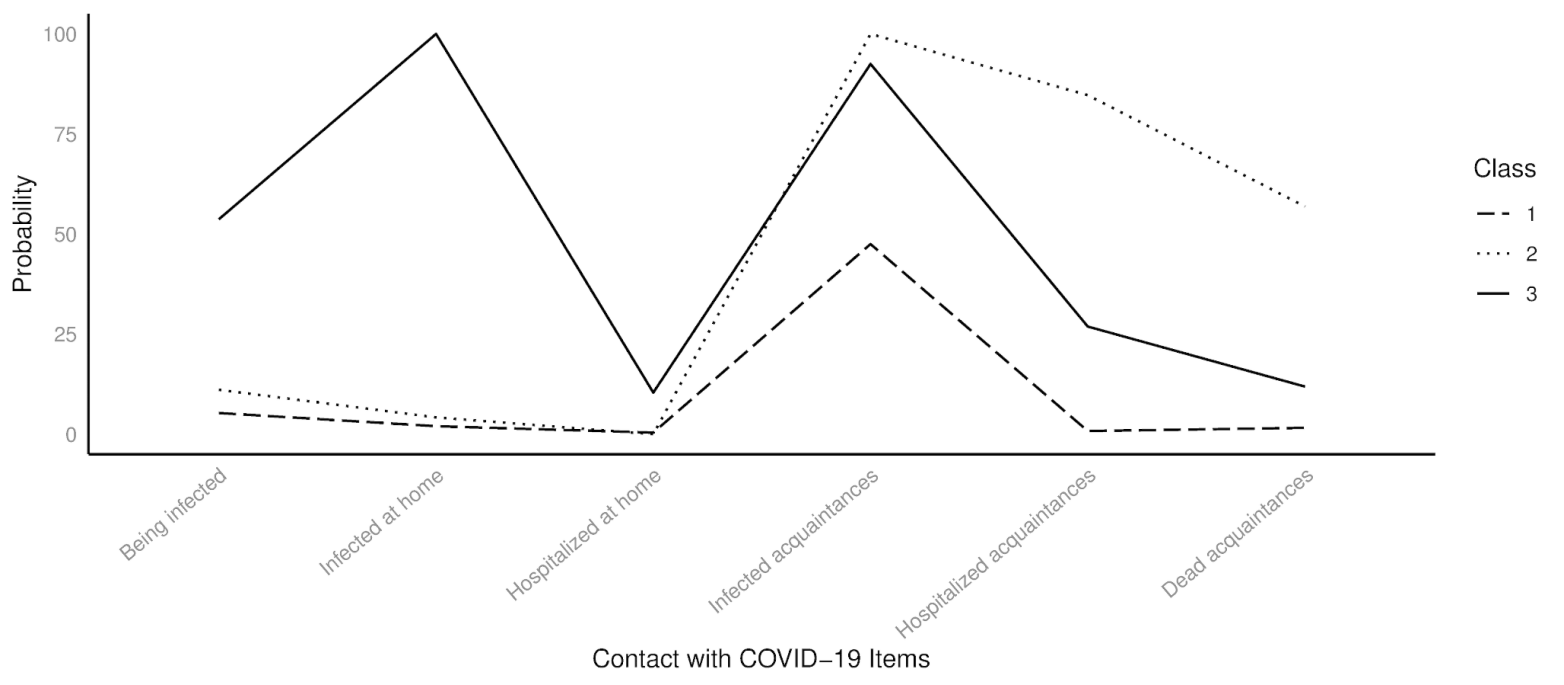
Contact with COVID-19 profiles. The X-axis represents the item number and the Y-axis represents the probability of answering “yes” to the given item, given that you belong to a particular contact with the COVID-19 class. The three COVID-19 contact classes are represented as the three different lines: (1) No/little contact, (2) Acquaintances, and (3) At Home

Table 3 displays the fit indices for the classes’ solutions. Two solutions displayed adequate entropy (three- and four-classes). From these, the three-classes solution was considered preferable because it obtained a lower BIC value than the four-classes solution (BIC value difference = 15). According to Raftery (1995) [48], a BIC value difference ≥ 10 is considered very strong evidence. Moreover, the four-classes solution included a class consisting of only 25 adolescents, which was quite similar to C1. Consequently, we selected the three-classes model as the most appropriate for this study’s sample. Figure 1 displays the graphic representation of these classes. As can be seen, three classes can be described as follows: (1) No contact at all or just knowing someone (friend, family, or acquaintance outside home) with COVID-19; (2) Knowing someone outside the home (friend, family, or acquaintance) who has been infected, hospitalized, and/or died from COVID-19; and (3) Being infected or living with someone who has been infected and/or hospitalized with COVID-19. Figure 1 shows contact with COVID-19 across the three latent classes. The three classes comprised 63%, 19.3%, and 17.7% of the participants, respectively.

**Table 3:** Results of Latent Class Analyses

Fit Statistics	One-class model	Two-classes model	Three-classes model	Four-classes model
AIC	1986	1843	1794	1781
BIC	2010	1894	1873	1888
SSABIC	1991	1853	1809	1802
Entropy		0.66	0.80	0.81
Bootstrapped likelihood ratio test		< 0.001	< 0.001	< 0.001
Sample size of each class	C1 = 384	C1 = 241	C1 = 244	C1 = 63
		C2 = 143	C2 = 72	C2 = 69
			C3 = 67	C3 = 25
				C4 = 227
*Note*. AIC = Akaike Information Criterion; BIC = Bayesian Information Criterion; SSABIC = Sample-Size Adjusted BIC; C = class				

Table 1 shows the descriptive statistics for all the study variables at W1 and W2 for each class. In Class 1, there were no significant differences at W1 compared to W2 in internalizing symptoms, *t*(210) = -0.33, *p* = .743, or DM, *t*(211) = 0.51, *p* = .608. There was a significant difference in internalizing symptoms, with higher levels at W2 compared to W1 in Class 2 (Acquaintances), *t*(58) = -3.44, *p* = .001, *d* = 0.37, and in Class 3 (At Home), *t*(50) = -2.24, *p* = .029, *d* = 0.35. Specifically, from W1 to W2 there was an increase in depressive symptoms in Class 2, *t*(58) = -3.60, *p* = .001, *d* = 0.47, and Class 3, *t*(50) = -2.23, *p* = .031, *d* = 0.35; an increase in anxiety symptoms in Class 2, *t*(58) = -2.80, *p* = .007, *d* = 0.40, and Class 3, *t*(50) = -2.35, *p* = .023, *d* = 0.43; and an increase in stress symptoms in Class 2 *t*(58) = -2.29, *p* = .020, *d* = 0.30. DM levels were lower at W2 compared to W1 in Class 2, *t*(51) = 3.76, *p* < .001, *d* = − 0.50, and marginally significant in Class 3, *t*(58) = 1.96, *p* = .055.

## Predictive Associations between Variables

Two dummy variables were created to include the classes of contact with COVID-19 in the analyses: “Acquaintances” (coded as 1 if they belong to the second class and with 0 if not) and “At home” (coded as 1 if they belong to the third class). Class 1 was used as the reference group. The interaction terms for the classes and W1 DM were created using *z* scores in the DM variable: Acquaintances x DM and At home x DM.

**Fig. 2:**
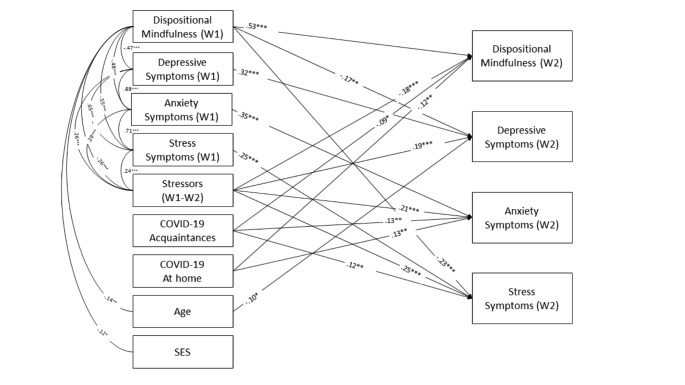
Statistically significant paths of the predictive model between internalizing symptoms, dispositional mindfulness, stressors and contact with COVID-19. Standardized values are displayed. *p < .05. **p < .01. ***p < .001

In path analysis, the predictive model displayed the following fit indexes: χ^2^(32, *N* = 383) = 169.835, *p* < .001, RMSEA = 0.106, 90% CI [0.091, 0.122], CFI = 0.925, TLI = 0.767, SRMR = 0.080. DM did not moderate the association between contact with COVID-19 and internalizing symptoms. A more parsimonious model was estimated excluding the interaction terms, with excellent fit indexes: χ^2^(16, *N* = 383) = 32.990, *p* = .007, RMSEA = 0.053, 90% CI [0.026, 0.078], CFI = 0.990, TLI = 0.952, SRMR = 0.034. This model explained 42, 33, 33, and 35% of the variance in DM, depression, anxiety, and stress symptoms at follow-up. Figure 2 shows the cross-lagged regressive coefficients of the model that were statistically significant and W1 covariances among DM, internalizing symptoms, stressors, age and SES. All the autoregressive paths were statistically significant, indicating the stability of those variables over the one-year follow-up. DM negatively correlated with stressors, internalizing symptoms (depression, anxiety and stress), age and SES; internalizing symptoms and stressors were positively correlated. DM predicted fewer depressive and stress symptoms prospectively. Stressors predicted more internalizing symptoms (depression, anxiety and stress) and less DM. Internalizing symptoms did not predict DM. Age negatively predicted depressive symptoms at W2. When controlling for previous internalizing symptoms, DM, stressors, age and SES, contact with COVID-19 (both At home and Acquaintances classes) predicted less DM and more anxiety symptoms prospectively. However, only Class 2 (Acquaintances) predicted more stress symptoms at W2, and none of the profiles directly predicted depressive symptoms prospectively.

## Mediation Analysis

The above findings suggested that DM could mediate the association between COVID-19 profiles and internalizing symptoms (depression and stress symptoms). We tested the four possible mediations by performing a bootstrapping analysis. The 95% confidence intervals for possible mediating effects are presented in Table 4. As can be seen, all of the confidence intervals for the indirect effects supported significant mediation effects, as they did not include zero. However, the total effects only were significant for Class 2 (Acquaintances), and the total effect was not significant for Class 3 (At Home). Moreover, there was a significant direct effect on stress of the COVID-19 contact Class 2 (Acquaintances). DM mediated contact with COVID-19 profiles (both Acquaintances and At Home) and depression and stress symptoms. Contact with COVID-19 Class 2 and Class 3 predicted lower levels of DM, which, in turn, predicted higher levels of depression and stress symptoms.

**Table 4:** Bootstrapping Direct, Indirect and Total Effects and 95% Confidence Interval (CI) (5000 Bootstrap Samples)

	*B*	Estimate	Std. Error	z-value	*p*	95% CI	
						Lower	Upper
**Direct Effects (c’)**							
COVID-19 Acquaintances◊ Depression	0.083	0.164	0.085	1.936	0.053	-0.008	0.329
COVID-19 At Home◊ Depression	0.060	0.122	0.094	1.299	0.194	-0.068	0.307
COVID-19 Acquaintances◊ Stress	0.121	0.209	0.075	2.799	0.005	**0.063**	**0.355**
COVID-19 At Home◊ Stress	0.068	0.122	0.085	1.422	0.155	-0.054	0.282
**Indirect Effects (a x b)**							
COVID-19 Acquaintances ◊ Dispositional Mindfulness ◊ Depression	0.015	0.029	0.018	1.605	0.108	**0.003**	**0.077**
COVID-19 At Home ◊ Dispositional Mindfulness ◊ Depression	0.020	0.040	0.022	1.838	0.066	**0.008**	**0.096**
COVID-19 Acquaintances ◊ Dispositional Mindfulness ◊ Stress	0.021	0.035	0.019	1.849	0.064	**0.004**	**0.081**
COVID-19 At Home ◊ Dispositional Mindfulness ◊ Stress	0.027	0.048	0.021	2.228	0.026	**0.014**	**0.098**
**Total Effects (c’ + ab)**							
COVID-19 Acquaintances◊ Depression	0.250	0.193	0.089	2.176	0.030	**0.014**	**0.363**
COVID-19 At Home◊ Depression	0.080	0.162	0.101	1.594	0.111	-0.044	0.359
COVID-19 Acquaintances◊ Stress	0.141	0.245	0.083	2.940	0.003	**0.084**	**0.408**
COVID-19 At Home◊ Stress	0.095	0.169	0.095	1.789	0.074	-0.027	0.345

## Sex Invariance of the Model

**Fig. 3:**
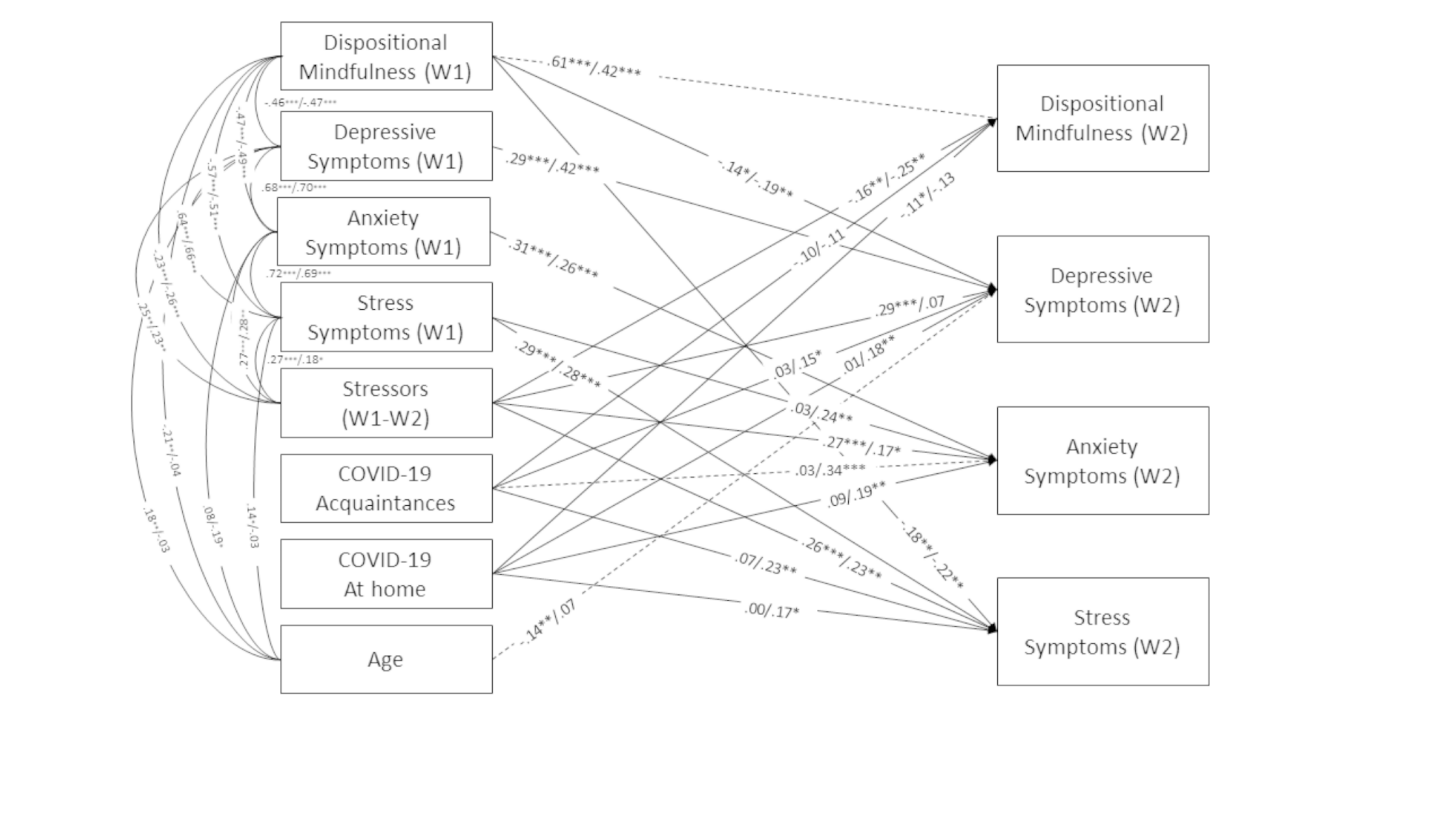
Paths in girls (left) and boys (right) of the predictive model (this model included the significant paths obtained for boys, girls, and the complete sample) between internalizing symptoms, dispositional mindfulness, stressors and contact with COVID-19. Standardized values are displayed (girls *n* = 222; boys *n* = 161). Dashed lines represent the paths that are significantly different in boys and girls. *p < .05. **p < .01. ***p < .001

Using multiple-group analysis, it was examined whether the above model was invariant in girls and boys. First, the original model was estimated separately for girls: χ2(32, *N* = 222) = 103.238, *p* < .001, RMSEA = 0.100, 90% CI [0.079, 0.122], CFI = 0.931, TLI = 0.787, SRMR = 0.079; and boys: χ2(32, *N* = 161) = 111.016, *p* < .001, RMSEA = 0.124, 90% CI [0.099, 0.149], CFI = 0.899, TLI = 0.686, SRMR = 0.100). Second, the configural invariance of the pattern of fixed and free model parameters across sex subsamples was determined (this model included the significant paths obtained for boys, girls, and the complete sample): χ2(32, *N* = 383) = 49.349, *p* = .026, RMSEA = 0.038, 90% CI [0.013, 0.057], CFI = 0.990, TLI = 0.976, SRMR = 0.044. This model showed improved fit indices in girls: χ2(32, *N* = 222) = 42.250, *p* = .106, RMSEA = 0.038, 90% CI [0.000, 0.066], CFI = 0.989, TLI = 0.975, SRMR = 0.049, and boys: χ2(32, *N* = 161) = 58.994, *p* = .003, RMSEA = 0.072, 90% CI [0.042, 0.101], CFI = 0.963, TLI = 0.910, SRMR = 0.067. Figure 3 displays the coefficients for girls and boys. Finally, we estimated a model in which longitudinal paths were constricted to be equal across both subsamples. This imposition did increase χ2 significantly, ∆χ2(20, *N* = 383) = 42.411, *p* = .002. Then, we examined individual paths to detect where the differences were. Three paths were significantly different in boys and girls. The DM autoregressive path from W1 to W2 was significantly higher in girls than in boys, χ2(1, *N* = 383) = 4.153, *p* = .042. There was a significant negative association between age and depressive symptoms (W2) in girls, but not in boys, χ2(1, *N* = 383) = 8.361, *p* = .004. There was a significant positive association between Acquaintances Covid-19 profile and anxiety symptoms (W2) in boys, but not in girls, χ2(1, *N* = 383) = 6.278, *p* = .012.

## Discussion

Adolescence is a vulnerable period for the development of negative mental health symptoms. Preliminary data since the onset of COVID-19 suggest that adolescents are at high risk of poor mental health outcomes due to the pandemic [4, 8]. However, longitudinal studies that include pre-pandemic data are scarce. Therefore, this study aimed to examine the impact of contact with COVID-19 on internalizing symptoms on Spanish adolescents and whether DM moderated this association, controlling for previous internalizing symptoms and other stressors different from COVID-19. Further, because stressful events, such as having a close contact with COVID-19, might have influenced adolescent’s own protective factors (as it is DM), the mediating role of DM between COVID-19 contact and internalizing symptoms was also explored. Additionally, classes of adolescents were identified based on their level of contact with COVID-19.

Three classes were identified according to adolescents’ level of contact with COVID-19: (1) No contact at all or just knowing someone (friend, family, or acquaintance outside home) with COVID-19; (2) Knowing someone outside the home (friend, family, or acquaintance) who has been infected, hospitalized, and/or died from COVID-19; and (3) Being infected or living with someone who has been infected and/or hospitalized with COVID-19. The results from this sample of Spanish adolescents showed that those who were more exposed to COVID-19 (i.e., Class 2 and 3) experienced a significant increase in internalizing symptoms before the pandemic (October 2019) compared to one year after (October 2020), and a decrease in DM. Specifically, the path analysis results indicated that contact with COVID-19 predicted an increase in internalizing symptoms (stress and anxiety), and a decrease in DM. Importantly, the results are not spurious due to other stressors different from the COVID-19, pre-pandemic internalizing symptoms, age or SES. The deterioration of mental health in adolescents following COVID-19 confirms previous reviews of cross-sectional studies suggesting a negative impact of COVID-19 on adolescents’ mental health [5, 8].

Specifically, the results of this study suggest that being infected or knowing someone close (both acquaintances and people adolescents lived with) who, during the first two waves of the pandemic, have been infected and/or hospitalized or even died from COVID-19 was associated with an increase in anxiety symptoms. Additionally, our results also suggested that knowing someone outside the home (friend, family, or acquaintance) who has been infected, hospitalized, and/or died from COVID-19 increased stress symptoms. Thus, having a closer contact with COVID-19 may increase risk perception, fear of COVID-19 infection, and perceived threat and uncertainty, all of which have been associated with negative mental health symptoms during the pandemic [16, 49–51]. Indeed, a study on Spanish adolescents found that fear of a family member or close friend being infected with COVID-19 was related to an increase in emotional difficulties [22]. Contrary to our expectations, depressive symptoms were not directly affected by closer contact with COVID-19. A possible explanation could be that, as our data was collected during the first two pandemic waves in Spain, the uncertainty and fear associated with these first moments might have been related to increased stress and anxiety. However, it could be that depressive symptoms would come later, when there is an accumulation of the negative consequences of the pandemic (e.g., losses of lives, work, and pandemic fatigue).

Results also indicated that DM longitudinally predicts fewer internalizing symptoms (depression and stress), as has been proposed in previous studies [13, 52]. However, contrary to expectations and earlier findings suggesting a protective role of DM in the face of stressors [13, 26], this study did not find that DM moderated the effect that contact with COVID-19 exerted on internalizing symptoms. Interestingly, COVID-19 contact negatively predicted DM, building on results from previous studies, which have found a negative association between worry and fear of COVID-19 and DM [27, 28, 30].

Furthermore, bootstrapping analysis supported the mediation role of DM between contact with COVID-19 and internalizing symptoms (stress and depressive symptoms). These results would suggest a direct effect between contact with COVID-19 and anxiety or stress, while the association between the contact with COVID-19 and depression only was indirect, through a decrease in DM. These results further support a previous study that found that the post-traumatic stress symptoms caused by experiencing a tornado longitudinally predicted less DM, which might make adolescents more susceptible to internalizing symptoms [29]. As these authors stated, it may be that experiencing a stressful situation reduced the adolescents’ ability to stay aware of the present moment, diminishing their ability to cope with the stressful situations and engage in more adaptive coping strategies, which may place them at higher risk of internalizing symptoms. Living on automatic pilot (as opposed to acting with awareness) may reduce awareness of thoughts and behaviors and thus lead to a failure to implement adequate emotion regulation strategies or engage in positive changes related to lifestyle or social support that could mitigate the negative impact of COVID-19, as suggested previously [53].

Finally, there were some differences between boys and girls in the predictive model. Specifically, the longitudinal association of DM was stronger in girls compared to boys; age was negatively related to depressive symptoms in girls but not in boys; and knowing someone outside the home (friend, family, or acquaintance) who has been infected, hospitalized, and/or died from COVID-19 predicted anxiety only in boys. However, these results should be taken with caution as the sample size for boys and girls was small for complex models, and results might not be replicable.

## Strengths and Limitations

A primary limitation of the current study is the small sample size and the attrition rate, which did not allow us to perform a more sophisticated analysis, such as structural equation modeling. However, to the best of our knowledge, this is one of the few studies that used longitudinal data with pre-pandemic data on Spanish adolescents and included a measure to control for other stressors different from COVID-19. Therefore, we could control for previous symptoms, as adolescence is known to increase internalizing symptoms [3], which might be a confounder. A second limitation is that this study only examined a very narrow aspect of COVID-19 impact (i.e., COVID-19 contact), and yet there are many other aspects of COVID-19 experiences not considered (e.g., parental job loss, isolation); therefore, future studies investigating other COVID-19 experiences would be very interesting. A third limitation is that our sample was from a school characterized mainly by medium SES, and individuals from lower SES backgrounds might have been underrepresented in our sample. As a study pointed out, adolescents from low socioeconomic backgrounds might have been more exposed to the negative consequences of the virus [54], and therefore our results cannot be generalized to adolescents with low socioeconomic backgrounds. In this vein, future studies should explore the impact of contact with COVID-19 in vulnerable populations, such as low SES, adolescents with previous mental health symptoms, or clinical samples. It has been suggested that the impact on COVID-19 might have been especially detrimental to individuals from vulnerable populations [4]. In addition, our sample only comprised adolescents from Spain, which is known as one of the leading global pandemic epicenters for COVID-19. Specifically, the sample was from Vitoria-Gasteiz, a city in the Basque country that is suspected to be where COVID-19 entered Spain in February 2020 [55]. Finally, since DM can improve with training, and although participants did not receive any training in mindfulness as part of this research, we cannot rule out the possibility that participants may have received some mindfulness training.

## Conclusions and Implications

This is the first study longitudinally examining the impact of contact with COVID-19, DM, and symptoms in adolescents, controlling for pre-pandemic levels of symptoms and the presence of other stressors. Our findings confirmed the negative impact of COVID-19 contact on adolescents’ internalizing symptoms. Although the sample consisted of adolescents who were not among the population groups at highest risk for developing severe cases of COVID-19, the results demonstrate the need to consider the negative consequences of the pandemic on this population’s mental health. Moreover, a deterioration in DM emerged as a mediating mechanism through which COVID-19 negatively affects adolescents’ mental health. Therefore, implementing mindfulness-based interventions at schools might be helpful for mediating the negative effect of the pandemic on adolescents.

## Summary

Adolescents are especially vulnerable to the mental health consequences of the COVID-19 pandemic. Longitudinal research that includes pre-pandemic data is needed to assess the impact of the pandemic in young populations. The main objective of this study was to examine the association between contact with COVID-19 and internalizing symptoms, controlling for pre-pandemic internalizing symptoms and other stressors in Spanish adolescents. Additionally, the moderation and mediation roles of DM were examined. Spanish adolescents (*N* = 383; 58% female; *M*age = 15.62) completed self-reported measures of DM and internalizing symptoms before and after the onset of the COVID-19, stressors, and contact with COVID-19. Three adolescent profiles emerged according to their contact with COVID-19: (1) little/no contact with COVID-19, (2) knowing someone close but outside the home who was infected, hospitalized, or died, and (3) being infected or someone at home being infected and/or hospitalized. Compared to little or no contact with COVID-19, both contact profiles longitudinally predicted internalizing symptoms, controlling for previous internalizing symptoms and other stressors different from COVID-19. Specifically, Class 2 and 3 were associated with anxiety, and Class 2 was associated with stress symptoms. DM mediated the association between contact with COVID-19 profiles and depression and stress symptoms: contact with COVID-19 predicted less DM in the long term, which in turn predicted an increase in depression and stress symptoms. This study suggests that contact with COVID-19 predicts increased internalizing symptoms in adolescents. In addition, this phenomenon could be partially explained by the decrease in the levels of adolescents’ DM. Mindfulness-based interventions could be beneficial for adolescents to cope with internalizing symptoms caused by contact with COVID-19.

## Data Availability

The datasets generated and analyzed during the current study are available in the Open Science Framework (OSF) repository (https://osf.io/xbwz9/).
